# Association Between Sex-Specific Serum Gamma-Glutamyltransferase and Incidence of Hypertension in a Chinese Population Without Metabolic Syndrome: A Prospective Observational Study

**DOI:** 10.3389/fcvm.2021.644044

**Published:** 2021-04-16

**Authors:** Xiaoyun Wu, Dongjie Liang, Junfang Sun, Yanyan Lin, Shengjie Wu

**Affiliations:** ^1^Department of Rehabilitation, The First Affiliated Hospital of Wenzhou Medical University, Wenzhou, China; ^2^Department of Cardiology, The First Affiliated Hospital of Wenzhou Medical University, Wenzhou, China; ^3^The Key Lab of Cardiovascular Disease of Wenzhou, Wenzhou, China; ^4^Department of Neurology, The First Affiliated Hospital of Wenzhou Medical University, Wenzhou, China

**Keywords:** gamma-glutamyltransferase, hypertension, metabolic syndrome, sex-specific, Chinese population

## Abstract

**Background:** Higher serum gamma-glutamyltransferase (GGT) is associated with high risk of hypertension. We aimed to examine the association between sex-specific serum GGT levels and incident of hypertension in a Chinese population without metabolic syndrome.

**Methods:** Participants who were free of hypertension and metabolic syndrome from the First Affiliated Hospital of Wenzhou Medical University between 2009 and 2014 were included. Participants were grouped into sex-specific quartiles of GGT levels (Q1–Q4) defined as: ≤19, 20–26, 27–38, and ≥39 U/L for male; ≤12, 13–15, 16–19, and ≥20 U/L for female. Cox proportional hazards models were used to estimate hazard ratios (HRs) and 95% confidence intervals (CIs) for incidence of hypertension according to sex-specific quartiles of GGT levels. Kaplan–Meier analysis and interaction analysis were conducted.

**Results:** Among 38,806 participants included (average age 36.0 years, 54.0% men), 4,505 of them developed hypertension. In the overall study population, using Q1 as the reference group, participants in Q2, Q3, and Q4 showed a higher risk of developing hypertension, with HRs (95% CIs) of 1.126 (1.029–1.232), 1.187 (1.083–1.302), and 1.300 (1.182–1.431), respectively (*P* < 0.001), after adjusting for known confounders. Sex-specific analysis showed that the adjusted HRs for participants in Q4 (reference: Q1) were greater in females [1.321 (1.100–1.586, *P* < 0.001)] than in males [1.268 (1.133–1.420, *P* < 0.001)] (*P* for interaction = 0.047). Moreover, interaction analysis showed that this association was consistently observed when the participants were stratified by age, body mass index, and fatty liver status.

**Conclusion:** Among Chinese adults without metabolic syndrome, serum GGT level was positively associated with incidence of hypertension, and the association was stronger in females than in males.

## Introduction

Hypertension is a leading global health risk affecting at least one billion people worldwide (1). Despite considerable knowledge on how to prevent and treat hypertension, the prevalence of this condition is increasing due to population growth and aging (2, 3). Furthermore, the awareness, treatment, and control of hypertension are still limited in both rural and urban communities worldwide (4), making hypertension the largest contributor of global disability-adjusted life-years (5).

Serum gamma-glutamyltransferase (GGT), on the other hand, has received much attention in recent years due to its potential role in cardiovascular risk factors and diseases (6, 7). GGT is the enzyme responsible for the extracellular catabolism of glutathione (GSH), a major antioxidant agent in the body (8). Several epidemiological studies have suggested that a high serum GGT level has a connection with the development of hypertension and that even an increase in serum GGT level within the normal range is independently associated with the incidence of hypertension (9). However, some studies found that this association was not statistically significant (10, 11). In addition, since serum GGT level is positively associated with metabolic syndrome, there could be residual confounding from prevalent metabolic syndrome in the statistical analyses of previous studies. Also, adult males and adult females have different serum GGT levels, with males having higher levels than age-matched females (12). Several studies showed that the association between GGT levels and cardiometabolic diseases was stronger in males than in females (13, 14). However, evidence is lacking on the association between sex-specific serum GGT level and incidence of hypertension.

Therefore, we conducted this study to investigate the association between sex-specific serum GGT level and incidence of hypertension in Chinese adults without metabolic syndrome.

## Materials and Methods

### Study Design and Population

0 This is a prospective observational study. The sample comprised individuals who underwent an annual health checkup at the First Affiliated Hospital of Wenzhou Medical University from December 2009 to December 2014. Participants who were free of hypertension and metabolic syndrome at the first health checkup and who had received at least one repeat health examination were enrolled (*n* = 43,122). Among them, we excluded those who met the following exclusion criteria: missing data on GGT levels or components of metabolic syndrome (*n* = 3,181), missing data on fatty liver status (*n* = 1,056), and alanine aminotransferase (ALT) levels more than five-fold of the upper limit of the reference range (>40 U/L) (*n* = 79). Thus, we included a total of 38,806 participants for the final analysis.

Verbal informed consent was obtained from each participant before their participation in the study. In addition, the personal information of each participant was substituted by a health examination number. The study protocol was approved by the Ethics Committee of the First Affiliated Hospital of Wenzhou Medical University.

### Measurements and Definitions

All participants were asked to fast and avoid smoking and strenuous exercise at least 12 h before their examination. The examination included a physical examination by a doctor, anthropometry, blood pressure (BP) measurement, a blood draw procedure, and abdominal ultrasonography. With the participant not wearing shoes, height was measured to the nearest centimeter. Weight was measured with the participant in light clothing without shoes to the nearest 0.1 kg. Body mass index (BMI) was calculated conventionally. BP, including systolic BP (SBP) and diastolic BP (DBP), was measured using a noninvasive automated sphygmomanometer (OMRON, Japan) with the participant in a quiet environment and in a sitting position after a 5-min period of relaxation. The measurements were repeated twice at the same visit, and the average of the recorded measurements was used. Serum fasting plasma glucose (FPG), total cholesterol (TC), triglyceride (TG), high-density lipoprotein cholesterol (HDL-C), low-density lipoprotein cholesterol (LDL-C), creatinine (Cr), ALT, and aspartate aminotransferase (AST) levels were measured with an automated immunochemistry analyzer (Abbott AxSYM, Illinois, USA) using standard methods. Fatty liver status was defined as the occurrence of at least two of the following abnormal findings by ultrasonography: diffusely enhanced liver echogenicity greater than that of the spleen or kidney, vascular blurring, and deep attenuation of the ultrasound signal.

Prevalent hypertension was defined as SBP ≥ 140 mmHg or DBP ≥ 90 mmHg or use of antihypertensive medication at baseline. Metabolic syndrome was defined as the presence of three or more of the following risk factors (15): (1) TG ≥ 1.7 mmol/L, (2) HDL-C < 1.03 mmol/L for males and HDL-C < 1.29 mmol/L for females, (3) SBP ≥ 130 mmHg or DBP ≥ 85 mmHg or use of antihypertensive therapy, (4) FPG ≥ 5.6 mmol/L or previously diagnosed with type 2 diabetes, and (5) BMI ≥ 25 kg/m^2^ for both sexes. We substituted waist circumference with BMI as measurements of waist circumference were not obtained at baseline examination.

### Follow-Up for Incident Events

Participants were followed prospectively for any development of hypertension. The follow-up evaluations were performed annually during the observation period and consisted of procedures that were the same as those used during baseline examination. Incident hypertension was defined as (1) detection of high BP during health examination (measured BP > 140/90 mmHg) or (2) taking anti-hypertensive medication or (3) showing medical certificate of hypertension during the annual health visits.

### Statistical Analysis

Due to significant sex-specific differences in serum GGT level, participants were grouped into sex-specific quartiles of GGT levels, as follows: Q1: ≤19 U/L, Q2: 20–26 U/L, Q3: 27–38 U/L, and Q4: ≥39 U/L for males; and Q1: ≤12 U/L, Q2: 13–15 U/L, Q3: 16–19 U/L, and Q4: ≥20 U/L for females. The distribution of continuous variables was assessed, and natural log-transformations were applied for GGT, AST, and ALT levels. Descriptive data were expressed as means ± standard deviation or proportions and compared using the one-way analysis of variance (ANOVA) or chi-squared test, as appropriate. Cox proportional hazards models were used to estimate hazard ratios (HRs) and 95% confidence intervals (CIs) for risk of incident hypertension according to a per-unit increase in GGT level [natural log-transformed (log_e_)] and sex-specific quartiles of serum GGT levels. Model 1 was a univariate analysis for serum GGT level. Model 2 was adjusted for age and sex. Model 3 was adjusted for all confounding variables included in this study, namely age, sex, BMI, SBP, DBP, FPG, Cr, TC, TG, LDL-C, HDL-C, ALT, AST, and fatty liver status. To test for linear risk trends, quartile rank as a continuous variable was added in the regression models. Kaplan–Meier analysis was applied to calculate the cumulative hazard of incident hypertension during the follow-up. Curve-fitting using a generalized additive model was performed to assess potential nonlinear associations between GGT levels and the incidence of hypertension in males and females. Furthermore, stratified analyses were conducted to investigate whether the association between serum GGT level and incidence of hypertension varied by pre-specified factors, including sex, age, BMI, and fatty liver status. Effect modification by these factors was assessed by including an interaction term in the final model. The analyses were performed using SPSS Statistics version 24.0 (SPSS, IBM Corp., Armonk, NY, USA) and R software version 3.4.3 (R Foundation for Statistical Computing, Vienna, Austria). All *P*-values were two-sided, and statistical significance was defined at *P* < 0.05.

## Results

### Characteristics of Participants

A total of 38,806 participants were included in this study. Hypertension occurred in 4,505 participants (3,334 males and 1,171 females) during the follow-up period of approximately 5 years. Table 1 summarizes the baseline characteristics of the cohort in terms of serum GGT level stratified by sex. The incidence of hypertension increased from 13.6% in Q1 to 19.9% in Q4 for males and from 4.7% in Q1 to 10.8% in Q4 for females. Age, weight, BMI, SBP, DBP, FPG, TC, TG, LDL-C, ALT, and AST were significantly higher while height and HDL-C were significantly lower in participants who had high serum GGT levels. Notably, participants with higher serum GGT levels exhibited a higher prevalence of fatty liver.

**Table 1:** Baseline characteristics of included subjects.

**Characteristics**	**Quartiles of baseline GGT (U/L) of 20,944 males**				***P***
	**Q1 (≤19)**	**Q2 (20–26)**	**Q3 (27–38)**	**Q4 (≥39)**	
*N*	5,197	5,643	4,911	5,193	
Incident hypertension	705 (13.6%)	797 (14.1%)	799 (16.3%)	1,033 (19.9%)	<0.001
Age	39.2 ± 14.9	39.3 ± 14.1	40.3 ± 13.1	41.6 ± 11.8	<0.001
Height, cm	170.9 ± 6.0	170.9 ± 5.9	170.7 ± 5.8	170.4 ± 5.7	<0.001
Weight, kg	63.6 ± 8.1	65.7 ± 8.3	67.6 ± 8.4	69.3 ± 8.3	<0.001
BMI, kg/m^2^	21.8 ± 2.4	22.5 ± 2.5	23.3 ± 2.5	23.9 ± 2.4	<0.001
SBP, mmHg	118.3 ± 10.4	119.5 ± 9.9	120.3 ± 9.8	120.9 ± 9.1	<0.001
DBP, mmHg	72.4 ± 7.7	73.1 ± 7.6	74.0 ± 7.5	75.3 ± 7.3	<0.001
FPG, mmol/L	5.1 ± 0.7	5.1 ± 0.7	5.2 ± 0.8	5.3 ± 0.9	<0.001
TC, mmol/L	4.4 ± 0.8	4.6 ± 0.8	4.8 ± 0.8	5.1 ± 0.9	<0.001
TG, mmol/L	1.1 ± 0.6	1.3 ± 0.7	1.6 ± 0.9	2.0 ± 1.4	<0.001
HDL-C, mmol/L	1.3 ± 0.3	1.3 ± 0.3	1.3 ± 0.3	1.3 ± 0.3	<0.001
LDL-C, mmol/L	2.4 ± 0.6	2.5 ± 0.6	2.6 ± 0.6	2.7 ± 0.7	<0.001
Cr, mmol/L	93.4 ± 12.4	93.5 ± 12.6	93.9 ± 17.4	92.9 ± 20.1	0.028
ALT^*^	2.8 ± 0.4	2.9 ± 0.4	3.1 ± 0.4	3.5 ± 0.5	<0.001
AST^*^	3.0 ± 0.2	3.1 ± 0.2	3.1 ± 0.3	3.3 ± 0.3	<0.001
FL, *N* (%)	432 (8.3%)	954 (16.9%)	1,510 (30.7%)	2,367 (45.6%)	<0.001
**Characteristics**	**Quartiles of baseline GGT (U/L) of 17,862 Females**				***P***
	**Q1 (≤12)**	**Q2 (13–15)**	**Q3 (16–19)**	**Q4 (≥20)**	
*N*	4,596	4,577	4,427	4,262	
Incident hypertension	217 (4.7%)	218 (4.8%)	276 (6.2%)	460 (10.8%)	<0.001
Age	35.7 ± 10.2	36.3 ± 10.6	37.8 ± 11.6	42.0 ± 12.8	<0.001
Height, cm	159.7 ± 5.1	159.7 ± 5.0	159.5 ± 5.2	159.1 ± 5.4	<0.001
Weight, kg	52.1 ± 6.0	52.3 ± 6.2	52.8 ± 6.3	54.1 ± 6.8	<0.001
BMI, kg/m^2^	20.5 ± 2.1	20.6 ± 2.2	20.8 ± 2.3	21.5 ± 2.6	<0.001
SBP, mmHg	109.8 ± 11.1	110.7 ± 11.0	111.6 ± 11.3	113.6 ± 11.3	<0.001
DBP, mmHg	67.6 ± 7.9	68.1 ± 7.7	68.7 ± 7.8	70.0 ± 8.1	<0.001
FPG, mmol/L	5.0 ± 0.4	5.0 ± 0.4	5.0 ± 0.5	5.1 ± 0.6	<0.001
TC, mmol/L	4.4 ± 0.8	4.5 ± 0.8	4.6 ± 0.8	4.8 ± 1.0	<0.001
TG, mmol/L	0.8 ± 0.4	0.9 ± 0.4	1.0 ± 0.5	1.2 ± 0.7	<0.001
HDL-C, mmol/L	1.6 ± 0.3	1.6 ± 0.3	1.6 ± 0.3	1.6 ± 0.3	0.024
LDL-C, mmol/L	2.2 ± 0.6	2.2 ± 0.6	2.3 ± 0.6	2.5 ± 0.7	<0.001
Cr, mmol/L	67.1 ± 12.0	66.8 ± 8.9	66.9 ± 9.5	67.3 ± 15.1	0.140
ALT^*^	2.4 ± 0.3	2.5 ± 0.4	2.6 ± 0.4	2.9 ± 0.5	<0.001
AST^*^	2.9 ± 0.2	2.9 ± 0.2	3.0 ± 0.2	3.1 ± 0.3	<0.001
FL, *N* (%)	86 (1.9%)	136 (3.0%)	239 (5.4%)	598 (14.0%)	<0.001

*GGT, gamma-glutamyltransferase; BMI, body mass index; SBP, systolic blood pressure; DBP, diastolic blood pressure; FPG, fasting plasma glucose; TC, total cholesterol; TG, triglyceride; HDL-C, high-density lipoprotein cholesterol; LDL-C, low-density lipoprotein cholesterol; Cr, creatinine; ALT, alanine aminotransferase; AST, aspartate aminotransferase; FL, fatty liver*.

* *natural log-transformed*.

### Risk Factor Analysis for Incident Hypertension

Table 2 shows the HRs and 95% CIs for risk of incident hypertension according to the GGT quartiles and per-unit increase in log_e_ GGT level. In the overall study population, the unadjusted HRs for incident hypertension were 2.024 (95% CI: 1.864–2.197, *P* < 0.001) for Q4 vs. Q1 (with high serum GGT levels) and 1.880 (95% CI: 1.808–1.955, *P* < 0.001) for log_e_ GGT level. When adjusted for sex and age (Model 2), serum GGT level showed an attenuated HR of 1.815 (95% CI: 1.671–1.970, *P* < 0.001) in Q4 and an attenuated HR of 1.411 (95% CI: 1.346–1.479, *P* < 0.001) for a per-unit increase in log_e_ GGT level. Finally, after a progressive adjustment for other known confounding variables (Model 3), serum GGT level remained ignificantly associated with incidence of hypertension, showing an HR of 1.300 (95% CI: 1.182–1.431), and the *P*-value for the trend across GGT quartiles was 0.001, with HR of 1.141 (95% CI: 1.073–1.213, *P* < 0.001) for a per-unit increase in log_e_ GGT. Notably, the sex-stratified analysis showed that the adjusted HRs for Q4 vs. Q1 were stronger in females [1.321 (95% CI: 1.100–1.586, *P* < 0.001)] than in males [1.268 (95% CI: 1.133–1.420, *P* < 0.001)] (*P* for interaction = 0.047).

**Table 2:** Risk of incident hypertension according to baseline serum gamma-glutamyltransferase quartiles.

	**Overall population**		**Male**		**Female**		
	**HR (95% CI)**	***P***	**HR (95% CI)**	***P***	**HR (95% CI)**	***P***	***P* for interaction**
**Model 1**							
Per unit-log_e_(GGT)	1.881 (1.808–1.958)	<0.001	1.366 (1.295–1.441)	<0.001	2.177 (1.961–2.415)	<0.001	<0.001
Q1 (Reference)	1.000		1.000		1.000		<0.001
Q2	1.232 (1.127–1.347)	<0.001	1.226 (1.107–1.357)	<0.001	1.174 (0.972–1.416)	<0.001	
Q3	1.519 (1.391–1.659)	<0.001	1.424 (1.287–1.576)	<0.001	1.752 (1.466–2.094)	<0.001	
Q4	2.024 (1.864–2.197)	<0.001	1.726 (1.568–1.899)	<0.001	2.851 (2.425–3.351)	<0.001	
*P* for trend	<0.001		<0.001		<0.001		
**Model 2**							
Per unit-log_e_(GGT)	1.411 (1.346–1.479)	<0.001	1.348 (1.279–1.421)	<0.001	1.406 (1.252–1.578)	<0.001	0.003
Q1 (Reference)	1.000		1.000		1.000		0.033
Q2	1.242 (1.136–1.358)	<0.001	1.261 (1.139–1.396)	<0.001	1.079 (0.894–1.303)	0.426	
Q3	1.472 (1.348–1.608)	<0.001	1.436 (1.297–1.589)	<0.001	1.361 (1.138–1.628)	0.001	
Q4	1.815 (1.671–1.970)	<0.001	1.707 (1.551–1.879)	<0.001	1.607 (1.363–1.896)	<0.001	
*P* for trend	<0.001		<0.001		<0.001		
**Model 3**							
Per unit-log_e_(GGT)	1.141 (1.073–1.213)	<0.001	1.133 (1.058–1.213)	<0.001	1.174 (1.019–1.352)	0.027	0.044
Q1 (Reference)	1.000		1.000		1.000		0.047
Q2	1.126 (1.029–1.232)	<0.001	1.151 (1.038–1.276)	0.007	1.019 (0.844–1.232)	0.842	
Q3	1.187 (1.083–1.302)	<0.001	1.189 (1.068–1.323)	0.002	1.165 (0.970–1.399)	0.102	
Q4	1.300 (1.182–1.431)	<0.001	1.268 (1.133–1.420)	<0.001	1.321 (1.100–1.586)	0.003	
*P* for trend	<0.001		<0.001		<0.001		

*Model 1 is univariate analysis for serum GGT. Model 2 is adjusted for sex, age. Model 3 is adjusted for sex, age, body mass index, baseline systolic blood pressure, baseline diastolic blood pressure, fasting plasma glucose, total cholesterol, triglyceride, high-density lipoprotein cholesterol, low-density lipoprotein cholesterol, creatinine, alanine aminotransferase, aspartate aminotransferase, and fatty liver status. Sex-specific quartiles of serum GGT in male: Q1 (≤19), Q2 (20–26), Q3 (27–38), Q4 (≥39); in female: Q1 (≤12), Q2 (13–15), Q3 (16–19), and Q4 (≥20)*.

*GGT, gamma-glutamyltransferase; IHT, Incident Hypertension*.

*P for interaction: examine the effect modification on the association between GGT and IHT by sex*.

**Figure 1:**
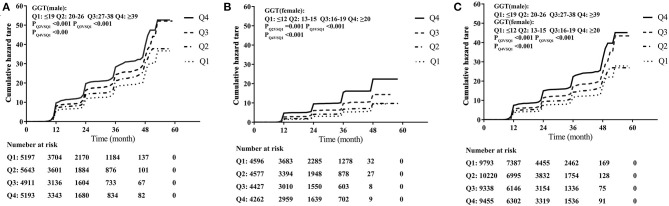
Incidence of hypertension stratified by sex-specific quartiles (Q) of serum gamma-glutamyltransferase (GGT) levels. **(A)** Incidence of hypertension in 20,944 males. **(B)** Incidence of hypertension in 17,862 females. **(C)** Incidence of hypertension in a total of 38,806 participants.

**Figure 2:**
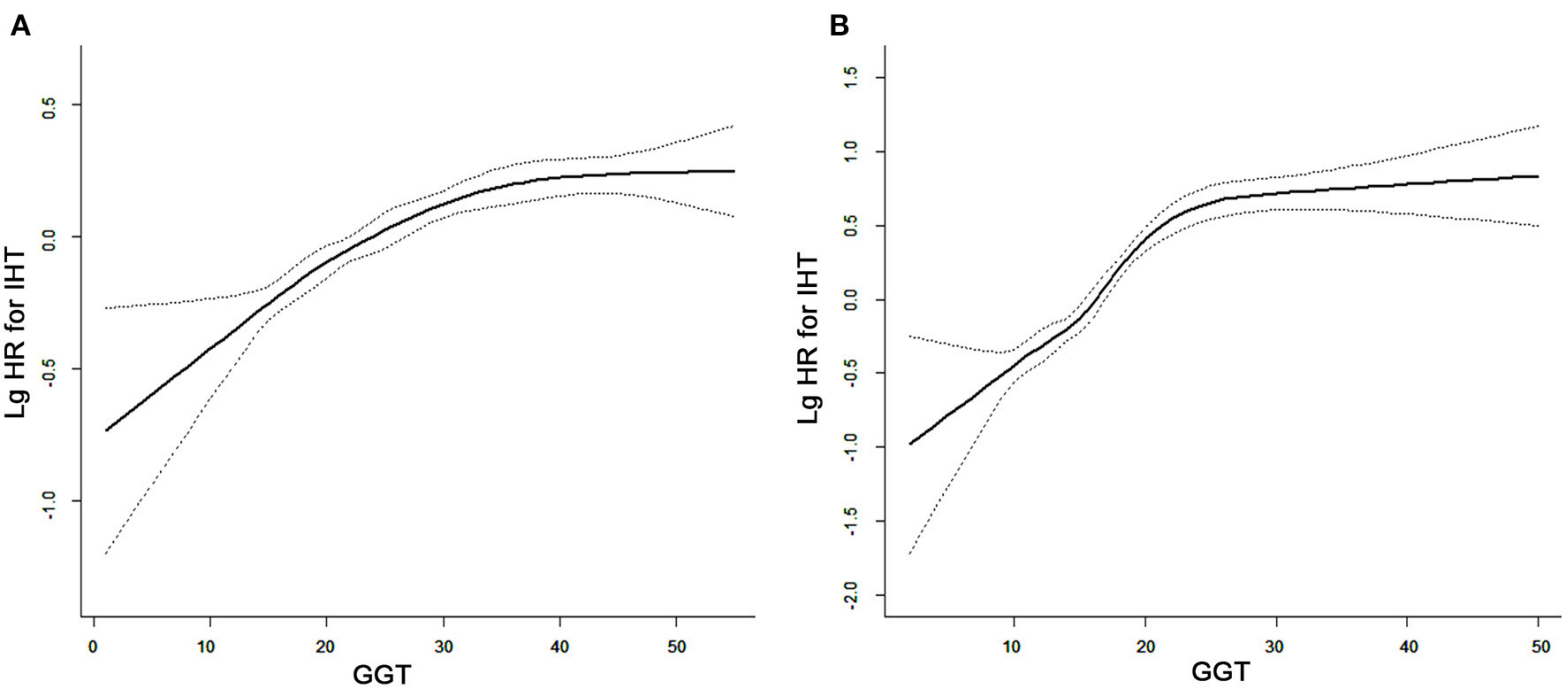
Curve-fitting between serum gamma-glutamyltransferase (GGT) level and incidence of hypertension in **(A)** males and **(B)** females.

Figure 1 demonstrates the unadjusted cumulative incidence of hypertension according to sex-specific quartiles of serum GGT levels. Baseline serum GGT level was significantly associated with future incidence of hypertension. Besides, serum GGT level appeared to play a more important role in the development of hypertension, with a larger increase in incidence (Q4 vs. Q1) among females than among males. As shown in Figure 2, GGT levels demonstrated a positive but nonlinear association with the risk of hypertension. The magnitude of increase in hypertension incidence was significantly higher in those with lower baseline GGT levels. The reduction was observed in the increase in the incidence rate with a turning point of 24 and 22 U/L, respectively.

### Subgroup Analysis for Risk of Hypertension

**Figure 3:**
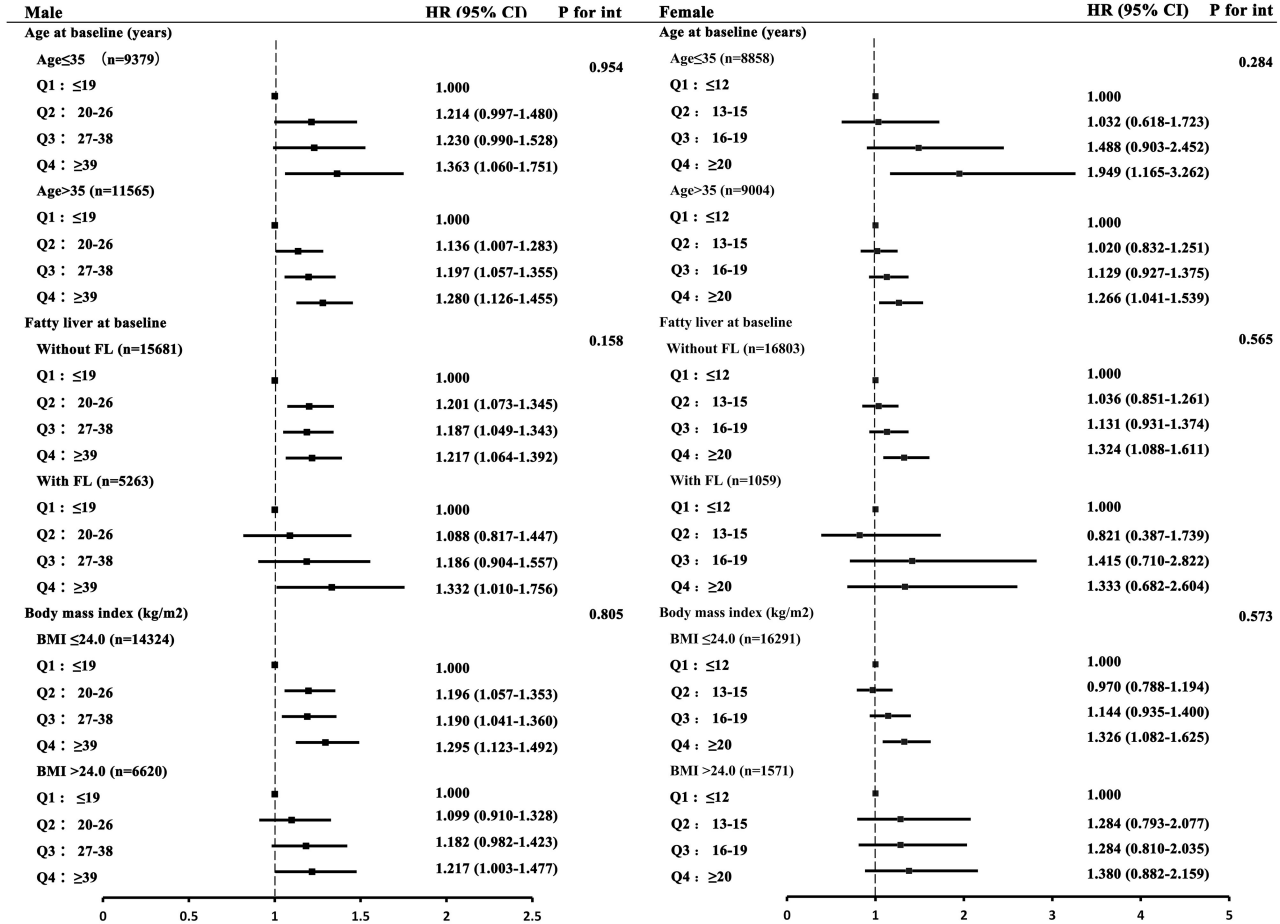
Forest plots of hazard ratios (HRs) with 95% confidence intervals (CIs) for quartiles of serum gamma-glutamyltransferase (GGT) levels stratified by sex. The values were adjusted for sex, age, body mass index (BMI), baseline systolic blood pressure, baseline diastolic blood pressure, fasting plasma glucose, total cholesterol, triglyceride, high-density lipoprotein cholesterol, low-density lipoprotein cholesterol, creatinine, alanine aminotransferase, aspartate aminotransferase, creatinine, and fatty liver (FL) status. *P* for int: *P*-values for the effect modification of age, FL status, and BMI on the association between serum GGT level and incidence of hypertension.

We tested the association between baseline serum GGT level and incidence of hypertension in clinically relevant subgroups according to age, BMI, and fatty liver status, and the results revealed a consistent positive association between the highest category of serum GGT level and risk of hypertension (Table 3 and Figure 3). Additionally, to determine whether the relationships between serum GGT level and incidence of hypertension differed between males and females stratified by age, BMI, and fatty liver status, we present forest plots of HRs in Figure 3. For males, the associations between serum GGT level and risk of hypertension were observed among all subgroups, whereas for females the associations were not statistically significant between subgroups stratified by fatty liver status and BMI.

**Table 3:** Risk of incident hypertension according to baseline serum gamma-glutamyltransferase quartiles stratified by age, fatty liver, and body mass index.

	**Cases (*n*)**	**Q1**	**Q2**	**Q3**	**Q4**	**Per unit-log_**e**_(GGT)**
**Age at baseline (years)**						
≤35	975/18,237	Ref	1.189 (0.990–1.429)	1.249 (1.025–1.522)	1.443 (1.151–1.808)	1.134 (0.977–1.316)
>35	3,530/20,569	Ref	1.115 (1.005–1.237)	1.188 (1.070–1.319)	1.294 (1.164–1.439)	1.166 (1.090–1.248)
**Fatty liver at baseline**						
Without	3,233/32,484	Ref	1.167 (1.058–1.287)	1.177 (1.060–1.305)	1.270 (1.139–1.417)	1.135 (1.054–1.222)
With	1,271/6,322	Ref	0.953(0.749–1.213)	1.177 (0.941–1.471)	1.272 (1.017–1.591)	1.134 (1.014–1.268)
**Body mass index (kg/m^2^)**						
BMI≤24.0	2,915/30,615	Ref	1.136 (1.022–1.263)	1.174 (1.051–1.312)	1.316 (1.173–1.476)	1.152(1.067–1.243)
BMI>24.0	1,590/8,191	Ref	1.133 (0.951–1.351)	1.220 (1.028–1.448)	1.275 (1.069–1.521)	1.111 (1.003–1.231)

*Adjusted for sex, age, body mass index, baseline systolic blood pressure, baseline diastolic blood pressure, fasting plasma glucose, total cholesterol, triglyceride, high-density lipoprotein cholesterol, low-density lipoprotein cholesterol, creatinine, alanine aminotransferase, aspartate aminotransferase, creatinine, and fatty liver status*.

*GGT, gamma-glutamyltransferase; FL, Fatty liver; BMI, body mass index; p int, p for interaction*.

## Discussion

In this large population-based study, we found that a higher GGT level was associated with an increased risk of incident hypertension in both males and females, even after further adjustment for age, sex, BMI, fatty liver status, and other established risk factors. Furthermore, the associations remained in a subsequent subgroup analysis stratified by age, BMI, and fatty liver status. No evidence of interaction occurred between GGT level and pre-specified factors except for sex. These findings thus suggest that GGT level is associated with the incidence of hypertension in males and especially in females.

Our results are consistent with those of previous studies (9, 16–18). In a prospective cohort study that included 4,704 normotensive young adults (age 18–30 years), Lee et al. (9) suggested that serum GGT level was a strong predictor of incident hypertension. Similarly, after analyzing the longitudinal data of 401 indigenous Australian adults, Li et al. (19) found that a higher serum GGT level was significantly associated with risk of hypertension independent of age, sex, BMI, and self-reported alcohol consumption. Notably, in a systematic review and dose-response meta-analysis that included 14 cohort studies, Kunutsor et al. (20) demonstrated that baseline GGT level was associated with a higher risk of hypertension in the general population and such association was consistent with a linear dose-response relationship. However, in those studies, sex-specific differences were not fully considered, and the sample sizes were relatively small. Moreover, the reported associations between serum GGT level and hypertension could have simply been confounded by a shared prevalence of metabolic syndrome. Therefore, our results further contribute to the current literature by (1) providing a large population-based longitudinal study of Chinese adults without metabolic syndrome and (2) demonstrating that the association between serum GGT level and incidence of hypertension may be determined for both males and females through sex-specific multivariate regression analysis.

Given the widely observed association between serum GGT level and risk of cardiometabolic disease (7, 21), several potential mechanisms have been proposed. First, GGT has a pro-oxidant effect during the catabolism of extracellular GSH. The hydrolysis of GSH catalyzed by GGT can lead to the generation of free radicals, lipid peroxidation, and mutagenesis, generally in the presence of iron (22). Thus, an elevated GGT level may identify individuals with a persistent increase in oxidative and cellular stress (7). In a 5-year longitudinal study, Ryoo et al. (23) demonstrated that serum ferritin, a highly sensitive indicator of body iron, was independently associated with impaired glucose tolerance, thereby highlighting an important clue regarding the above mechanistic pathway. Second, some studies suggested that an underlying fatty liver disease may represent the link between GGT level and hypertension (11, 24, 25). Previous studies have also shown that serum GGT level is positively associated with fatty liver (26–28). However, in our study, a significant association between serum GGT level and incidence of hypertension was still observed after adjusting for fatty liver status and other known confounding variables. In addition, further interaction analysis showed that the association between serum GGT level and risk of hypertension was not modified by fatty liver status. A similar finding was reported by Kim et al. (29) in a study investigating the association of GGT with risk of type 2 diabetes mellitus. Therefore, an underlying fatty liver disease may not account for the relationship between GGT level and incidence of hypertension. Third, elevated levels of serum GGT may reflect chronic subclinical inflammation (8), which was proposed as a key mechanism of hypertension (30). For example, the CARDIA study demonstrated that serum GGT level is associated with future levels of inflammation markers such as C-reactive protein or fibrinogen in a dose-response manner (9). This finding was also confirmed among 5,446 asymptomatic individuals in a recent study conducted by Ali et al. (31).

Few studies have evaluated sex-specific differences in the association between GGT and hypertension. In a meta-analysis, Kunutsor et al. (20) reported a stronger association between GGT level and risk of hypertension in males than in females, although the *P*-value for the meta-regression was >0.05. Notably, in our study, the relationship between elevated serum GGT level and incidence of hypertension was stronger in females than in males. Meanwhile, it has been reported that females have lower GGT activity than males due to the effect of sex hormones such as estradiol (32). In females, estradiol has an antioxidant effect, and an elevated serum GGT level may suggest a reduction in estradiol level (33). Thus, one possible mechanism for the sex-specific differences in the association between GGT and hypertension could be the effect of sexual hormones. However, this concept requires further investigation in appropriate animal and cell culture models. In summary, although the above mechanisms have been proposed, the exact pathway underlying the association between GGT and hypertension remains unknown. Thus, further studies investigating the role of GGT in hypertension are required.

Our findings highlight a clear and independent association between serum GGT level and risk of hypertension and can provide further insight into the prevention of hypertension. As serum GGT level may serve as a marker of oxidative stress and subclinical chronic inflammation (8), individuals with elevated serum GGT levels should be advised by doctors to pay attention to their health status and to measure their BP regularly. In addition, although the exact mechanism underlying this association remains uncertain, individuals with higher serum GGT levels may benefit from dietary and lifestyle modifications that can reduce the serum GGT level (34).

Although our findings are valid, our study has several limitations. The main limitation is the lack of anthropometric parameters regarding central obesity (i.e., waist/hip ratio), lifestyle (e.g., smoking, alcohol consumption), and dietary factors, which may be helpful to better understand the relationship between serum GGT level and incidence of hypertension. Another important limitation was that the diagnosis of hypertension was based only on measurements taken at a single visit; a dynamic analysis using values at different stages would yield more important and valuable results. Furthermore, the association that we observed was modest. However, if considered at the population level, such finding can have an important impact on public health. Given the epidemiological nature of our findings, the causality between serum GGT level and incidence of hypertension cannot be established. A randomized, multi-center, intervention study is needed in the future.

In conclusion, this study revealed that serum GGT level was an independent risk factor of future incident hypertension in Chinese adults without metabolic syndrome. Moreover, serum GGT level appeared to play a more crucial role in the development of hypertension in females than in males.

## Data Availability Statement

The raw data supporting the conclusions of this article will be made available by the authors, without undue reservation.

## Ethics Statement

The studies involving human participants were reviewed and approved by the First Affiliated Hospital of Wenzhou Medical University. Written informed consent for participation was not required for this study in accordance with the national legislation and the institutional requirements.

## Author Contributions

XW and DL: data collection and analysis and drafted the manuscript. JS and YL: data collection. SW: conceived the study, participated in its design, study supervision, obtained funding, and helped to draft the manuscript. All authors contributed to the article and approved the submitted version.

## Conflict of Interest

The authors declare that the research was conducted in the absence of any commercial or financial relationships that could be construed as a potential conflict of interest.
